# Characterization of class 1 integrons in metallo-β-lactamase-producing *Acinetobacter baumannii* isolates from hospital environment

**DOI:** 10.1186/s13104-023-06646-y

**Published:** 2023-12-09

**Authors:** Farzaneh Firoozeh, Mohammad Ghorbani, Mohammad Zibaei, Farzad Badmasti, Malihe Farid, Narges Omidinia, Fatemeh Bakhshi

**Affiliations:** 1 Department of Microbiology, School of Medicine, Alborz University of Medical Sciences, Karaj, 3149779453 Iran; 2 Evidence- Based Phytotherapy and Complementary Medicine Research Center, Alborz University of Medical Sciences, Karaj, Iran; 3 Department of Parasitology and Mycology, School of Medicine, Alborz University of Medical Sciences, Karaj, Iran; 4 Department of Bacteriology, Pasteur Institute of Iran, Tehran, Iran; 5 Social Determinants of Health Research Center, Alborz University of Medical Sciences, Karaj, Iran

**Keywords:** *Acinetobacter baumannii*, Hospital environment, metallo-β-lactamase, Integrons

## Abstract

**Background and Objective:**

The emergence and widespread dissemination of antibiotic resistance in *A. baumannii*, has become a globally challenge. The increasing hospital outbreaks by multi-drug resistant (MDR) *A. baumannii* strains, shows the necessity of continuous monitoring to find sources of resistant strains in hospitals. This study aimed to identify the presence of class 1 integrons and metallo-β-lactamase (MBL) related genes in *A. baumannii* isolates from hospital environment.

**Methods:**

In order to identify *A. baumannii* isolates, a total of 297 environmental samples were collected from burn wards and intensive care units (ICUs) of two university hospitals. Resistance to common antibiotics was studied by disk diffusion method and microbroth dilution assay was used to determine the minimum inhibitory concentrations (MICs) of imipenem, colistin and tigecycline. The *A. baumannii* isolates were studied by polymerase chain reaction (PCR) for the presence of class 1 integrons (*intI1*, *intl CS*) and metallo-β-lactamases (MBLs) (*bla*_IMP_, *bla*_VIM_, *bla*_NDM_) genes.

**Results:**

*A. baumannii* was identified in 68/297 (22.9%) of hospital environment. All *A. baumannii* strains were multidrug-resistant (MDR), but none of them were resistant to colistin, tigecycline and ampicillin-sulbactam. All (100%) and 38 (95.0%) of *A. baumannii* isolates from ICUs and burn wards were imipenem resistant respectively. Class 1 integrons was identified in 30/40 (75.0%) and 23/28 (82.1%) isolates from burn wards and ICUs respectively. Two different types of gene cassettes were identified, which included: *arr-2*, *ereC*, *aadA1*, *cmlA5* and *arr2*, *cmlA5.* MBL genes including *bla*_VIM_ and *bla*_IMP_ were detected in 26/28 (92.8%), 27/28(96.4%) and 39/40 (97.5%) and 31/40 (77.5%) of the isolates from the ICUs and the burn wards respectively. None of the isolates contained the *bla*_NDM−1_ gene.

**Conclusion:**

The findings of the present study showed that the isolation rate of MBL producing carbapenem-resistant *A. baumannii* (CRAB) was relatively high in the environmental surface of burn wards and ICUs, which can be considered as a potential source of outbreaks in hospitalized patients.

## Introduction

Carbapenem-resistant *Acinetobacter baumannii* (CRAB) is considered as a dangerous hospital pathogen [1]. The feature is related to its ability to persist in the hospital environment and acquire antibiotic resistance genes [2]. The environment is the main reservoir of *A. baumannii* and acts as a source of infection. *A. baumannii* persists for long periods of time in all hospital environments, including dry surfaces with limited nutrients. Some studies indicate that up to 48% of environmental surface are contaminated with *Acinetobacter* [3, 4]. The patient surrounding environmental sites that have a high probability of contamination include: patient’s bed, bed rails, bed sheets, and medical equipments such as ventilators, respiratory monitors as well as the hands of healthcare workers [5]. Hospitalized patients, especially intensive care units (ICUs) patients, are at high risk of acquiring multi-drug resistant (MDR) *A. baumannii* directly from contaminated environmental surface [6]. The MDR *A. baumannii* strains are defined as resistance to three or more antimicrobial classes [7]. The critical role of environmentally contaminated surfaces such as supply carts, floors, infusion pumps, and ventilator touch pads in the transmission of *A. baumannii* to patients has been demonstrated in previous studies [8, 9]. The long-term persistence of *A. baumannii* on hospital surfaces is also due to the high potential of acquiring antibiotic resistance genes [10]. *A. baumannii* has the ability to develop resistance against a wide range of antibiotics, including carbapenems, through various mechanisms [11]. The growing rate of resistance to carbapenems and other treatment options such as colistin in *A. baumannii* strains in the world is alarming [12]. The most important mechanism of resistance to carbapenems is enzymatic hydrolysis, which is carried out by enzymes called carbapenemases [13].

The Ambler class B β-lactamases includes zinc-dependent β-lactamases called metallo-β-lactamase (MBL), which are among the most important carbapenemases in *A. baumannii* strains [14]. The most effective MBLs with wide distribution in *A. baumannii* strains include VIM and IMP [15]. New Delhi β-lactamase (NDM) is also a MBL that has been reported in human and environmental isolates of *A. baumannii* in most regions of the world [16]. The high potential of metallobetalactamases for widespread expansion is related to the association of their coding genes with transferable genetic elements, including integrons [17, 18]. Integrons are mobile genetic elements with unique characteristics which commonly carry the cassettes containing the antimicrobial resistance genes. The most common integrons found in *A. baumanni*i strains, are classes 1 and 2, which play a fundamental role in antibiotic resistance and commonly encode β-lactamases and metallo-β-lactamases genes, followed by genes for resistance to chloramphenicol, and aminoglycoside and trimethoprim classes [19]. The association of MBL genes and integrons, in clinical strains of *A. baumannii* has been shown in many studies [15]. Identifying of *A. baumannii* sources in the hospital environment strengthens our knowledge about potential routes of *A. baumannii* transmission and helps to adopt more appropriate control policies against the spread of infection caused by this bacterium among patients. Therefore, the present study aimed to characterize class 1 integrons in metallo-β-lactamase-producing *Acinetobacter baumannii* isolates from the hospital environment.

## Materials and methods

### Samples

A total of 297 environmental samples, were collected from burn wards and ICUs of two university hospitals [Shahid Motahari Hospital (*n* = 240) and Shahid Madani Hospital (*n* = 57)] between April and September 2021. For sample collection moistened sterile cotton swabs were used, which was rolled over the surfaces. The collected environmental swabs were individually transferred to Brain Heart Infusion broth (BHI, Merck, Germany) media and incubated overnight at 37^o^C. Then the swabs were cultured on MacConkey agar and blood agar (Merck, Germany) plates at 37^o^C for 24 h. *A. baumannii* isolates were identified by standard biochemical tests and confirmed using PCR amplification of the *rpoB, bla*_OXA-51_ and *gluconolactonase* genes [15, 20].

### Antimicrobial susceptibility

Resistance patterns of *A. baumannii* isolates were determined by Kirby-Bauer disk diffusion test in accordance with Clinical and Laboratory Standard Institute (CLSI) [21], using antibiotics disks: imipenem (10 µg), ceftazidime (30 µg), ampicillin-sulbactam (20 µg), doxycycline (30 µg), ciprofloxacin (5 µg), gentamicin (10 µg), minocycline (30 µg), and trimethoprim/sulphamethoxazole (1.25/23.75 µg) (Mast, UK) [6]. The *A. baumannii* isolate was defined as MDR if it was resistant to three or more antimicrobial classes.

The minimum inhibitory concentrations (MICs) of imipenem, colistin and tigecycline were determined by microbroth dilution method. Susceptibility interpretation was done according to antimicrobial breakpoints organized by CLSI and European Committee on Antimicrobial Susceptibility Testing (EUCAST) [14]. Furthermore, the FDA breakpoints for susceptible (MIC ≤ 2 µg/mL), intermediate (4 µg/mL), and resistant (MIC ≥ 8 µg/mL) were used to categorize tigecycline susceptibility. *Escherichia coli* ATCC 25,922 and *Pseudomonas aeruginosa* ATCC 27,853, were obtained from Pasteur Institute of Iran, used as the quality control strains.

### Detection of class 1 integrons and MBLs genes

The presence of class 1 integrons genes (*intI1*, *intl CS*) and MBLs genes (*bla*_IMP_, *bla*_VIM_, *bla*_NDM− 1)_ was assessed by PCR using specific primers (Table 1) [15, 20, 22]. For this purpose, genomic DNA was extracted from all *A. baumannii* isolates by boiling method [6]. The total volume of the PCR reaction mixture was 25 µL, and PCR amplification was conducted in PCR thermal cycler (Eppendorf master cycler®, MA). PCR cycling conditions for amplification of class 1 integrons genes including *intI1*, and *intl CS* were: 1 cycle of (94ºC for 5 min), 40 cycles of (94ºC for 30 s, 58ºC for 30 s and 72ºC for 30 s), followed with 5 min at 72ºC. The amplification programs of the *bla*_VIM_, and *bla*_IMP_ genes were as follows: one cycle of 95 °C for 5 min; 35 cycles of 95 °C for 45 s; 55 °C for 45 s and 72 °C for 1 min, ending with a final extension temperature of 72ºC for 10 min [15, 16, 23]. The thermal conditions of the PCR reaction for *bla*_NDM− 1_ gene were programmed as follows: initial denaturation at 94 °C for 10 min, 36 cycles of denaturation (94 °C, 30 s), annealing (52 °C, 40 s) and primer extension (72 °C, 50 s), with a final extension at 72 °C for 10 min [22]. The PCR products were visualized after electrophoresis on 1% gel agarose using UV transilluminator (Bio-Rad, UK). *A. baumannii* strains carrying the studied genes, which were previously confirmed by sequencing, were used as positive controls.

**Table 1:** Specific PCR primers for the detection of genes used in this study

Gene	Primer	Sequence (5’-3’)	Size (bp)/Annealing temp.	References
*bla*_OXA−51_	F	CTA ATA ATT GAT CTA CTC AAG TTA C	988/56.5	[15]
	R	GAA TAC TCC ATT TGA ACC ART GG		
*rpoB*	F	CTG ACT TGA CGC GTG A	1024/57.0	[15, 20]
	R	TGT TTG AAC CCA TGA GC		
*gluconolactonase*	F	TTG GAG AAT GCC CAA CTT GG	185/56.5	[20]
	R	CCC GTC TTC GAG CGC AAC		
*intI1*	F	CAG TGG ACA TAA GCC TGT TC	160/58.0	[15]
	R	CCC GAG GCA TAG ACT GTA		
*CS*	F	GGC ATC CAA GCA GCA AG	Variable/58.0	[15]
	R	AAG CAG ACT TGA CCT GA		
*bla*_VIM_	F	GAT GGT GTT TGG TCG CAT A	390/55.0	[15, 22]
	R	CGA ATG CGC AGC ACC AG		
*bla*_IMP_	F	GGA ATA GAG TGG CTT AAY TCT C	232/55.0	[15, 22]
	R	GGT TTA AYA AAA CAA CCA CC		
*bla*_NDM_	F	GGT TTG GCG ATC TGG TTT TC	621/52.0	[22]
	R	CGG AAT GGC TCA TCA CGA TC		

### Statistical analysis

The data were analyzed using the Statistical Package for Social Sciences software version 23 (SPSS, Inc.). Comparison between variables was done using Chi-square or Fisher’s exact tests. *P-*values ≤ 0.05 were considered statistically significant.

## Results

**Fig. 1:**
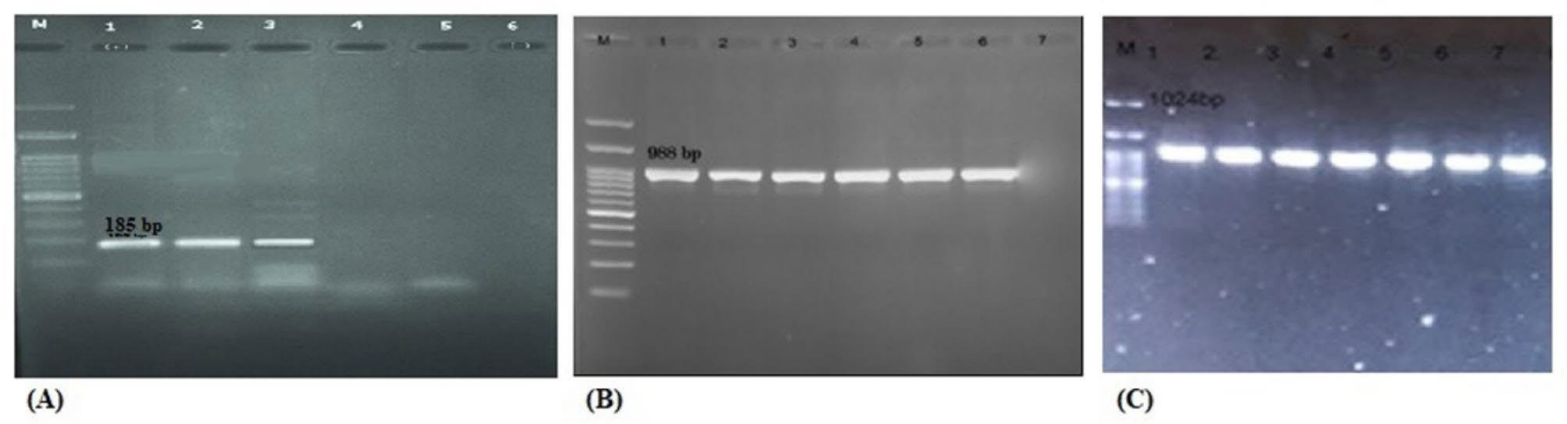
Amplification of (A) *gluconolactonase*, (B) *bla*_OXA−51_, and (C) *rpoB* genes. Lanes M: 100-bp DNA size marker; (A) Lane 1: positive control; lanes 2 and 3: *gluconolactonase* positive isolates; lane 4: negative control; (B) Lane 1: positive control; lanes 2–6: *bla*_OXA−51_ positive isolates; lane 7: negative control; (C) Lane 1: positive control; lanes: 2–7: *rpoB* positive isolates

Of 297 environmental samples collected, 53/240 (22.1%) *A. baumannii* isolates were identified in different environmental surface of Shahid Motahari Hospital in Tehran and 15/57 (26.3%) *A. baumannii* isolates from different environment of Shahid Madani Hospital in Karaj (Fig. 1). The hospital environment from which *A. baumannii* was isolated included the environmental surface of the ICUs and burn wards, which are separately shown in Table 2.

**Table 2:** * A. baumannii* isolates from environmental samples in burn wards and ICUs^†^

Source	Number	percent
**ICUs (N = 28)**		
Medical equipment Bed surfaces and bed rails	19 5	67.9 17.9
Door handles Other sites	2 2	7.1 7.1
**Burn wards (N = 40)**		
Medical equipment Bed surfaces and bed rails Bedside tables Door handles Computer Keyboard Nursing stations Mops	8 11 12 3 1 1 2 2	20.0 27.5 30.0 7.5 2.5 2.5 5.0 5.0

^†^ICUs: Intensive care units

**Fig. 2:**
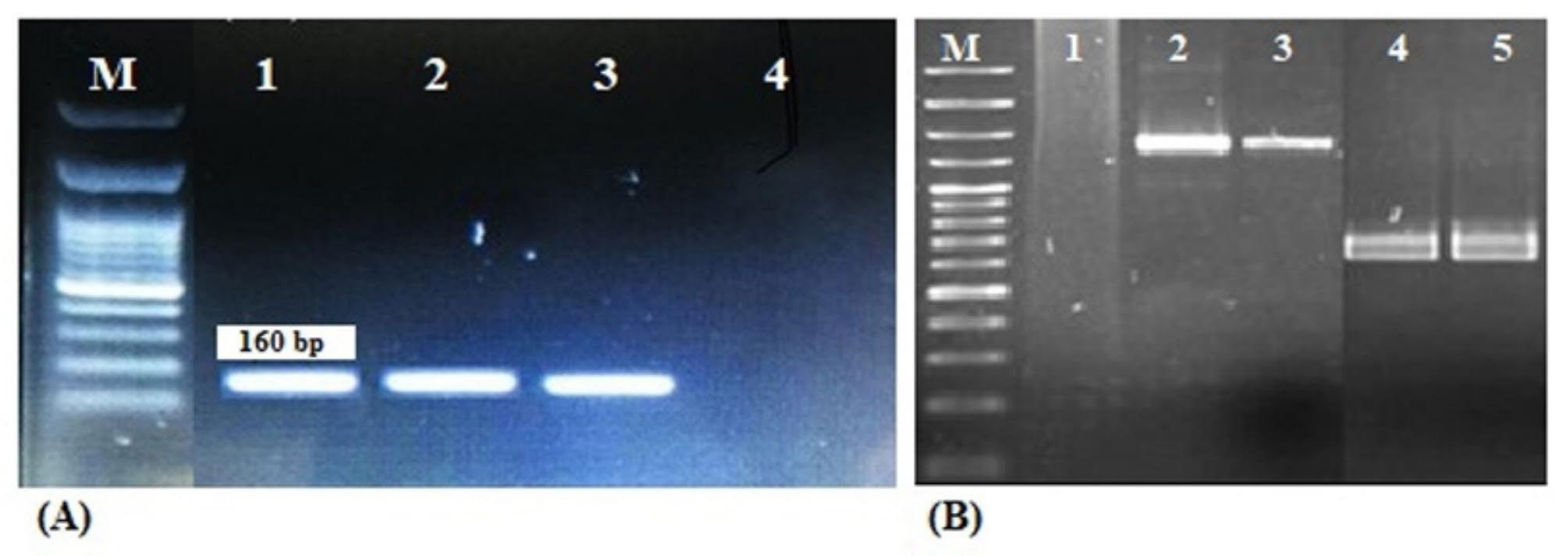
Electrophoresis of class I integron (A) *intI1* gene and (B) gene cassettes. Lanes M: 100-bp DNA ladder; (A) Lane 1: positive control; lanes 2 and 3: *intI1* positive isolates; lane 4: negative control; (B) Lane 1: negative control; lanes 2 and 3: isolates containing gene cassettes with a length of 1350 bp (*arr-2*, *ereC*, *aadA1*, *cmlA5*); lanes 4 and 5: isolates containing gene cassettes with a length of 700 bp (*arr-2*, *cmlA5*)

**Fig. 3:**
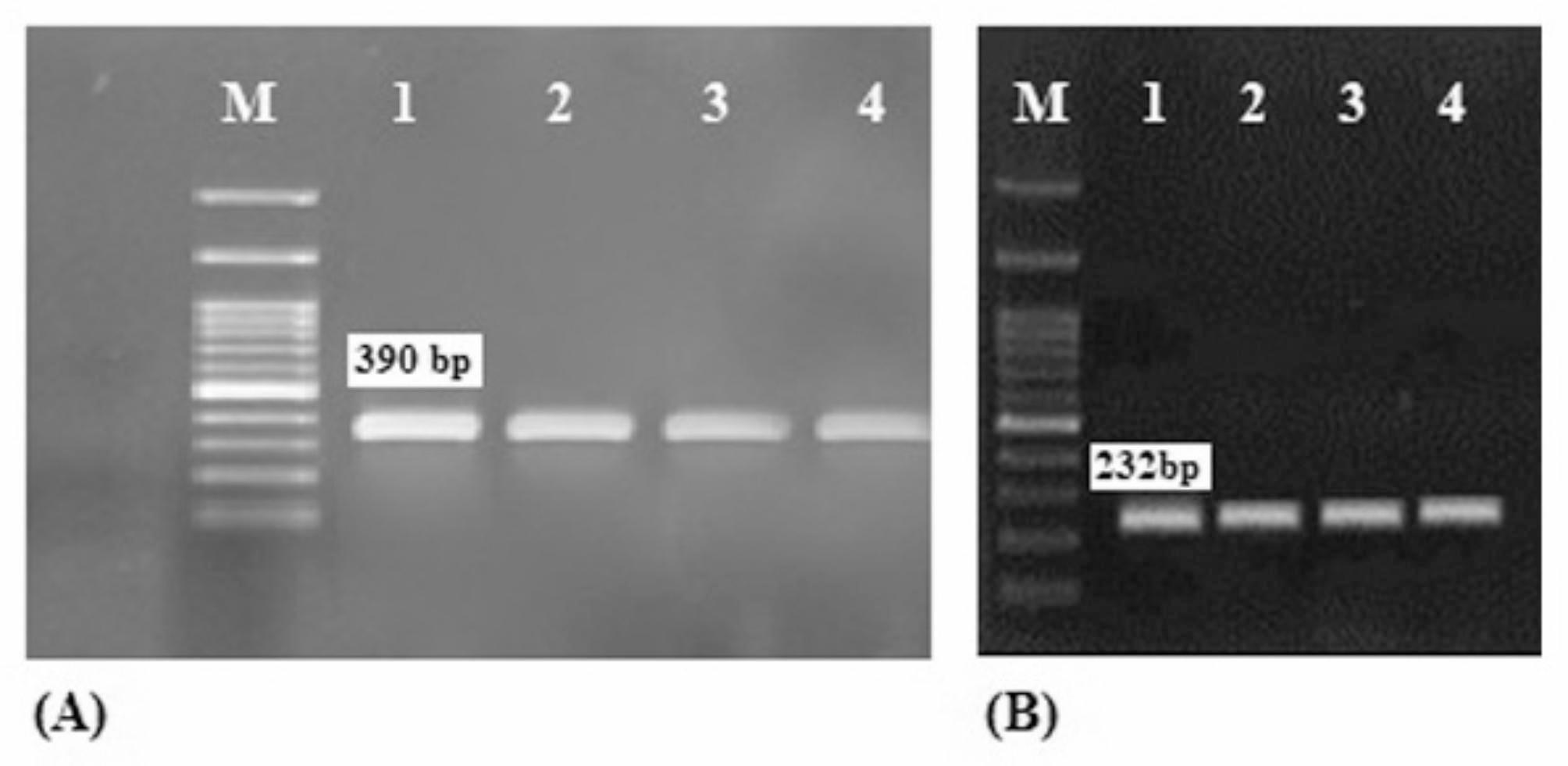
Electrophoresis of the PCR products for *bla*_VIM_ and *bla*_IMP_ genes. Lanes M: 100-bp DNA size marker; (A) Lane 1: positive control; lanes 2–4: *bla*_VIM_ positive isolates; (B) Lane 1: positive control; lanes 2–4: *bla*_IMP_ positive isolates

The results of resistance pattern investigation revealed all *A. baumannii* isolates from the hospital environment were MDR and showed resistance to three or more antimicrobial classes. In addition, none of the isolates were resistant to colistin, tigecycline and ampicillin-sulbactam. The highest level of resistance was obtained to trimethoprim/sulphamethoxazole and ceftazidime and 100% *A. baumannii* isolates from burn wards and ICUs were resistant to the mentioned antibiotics. In addition, resistance rates to imipenem in ICUs and burn wards were 100% and 95% respectively. All 28/28(100%), 33/40 (82.5%), 26/28(92.9%), 33/40 (82.5%) of *A. baumannii* isolates from ICUs and burn wards, were resistant to ciprofloxacin, and gentamicin respectively. The study of the resistance pattern also indicated that 4/28 (14.3%), 2/28 (7.1%) and 2/28 (7.1%) of *A. baumannii* isolates from ICUs were resistant to doxycycline, ampicillin-sulbactam and minocycline respectively, while none of the isolates from the burn wards were resistant to the mentioned antibiotics. Class 1 integrons integrase gene (*intI1*), was detected in 53/68 (77.9%) of *A. baumannii* isolates including 30/40 (75.0%) isolates from burn wards and 23/28 (82.1%) isolates from ICUs. All the *intI1*-positive *A. baumannii* strains were also positive for the *intl CS* gene (Fig. 2). Two types of gene cassettes were obtained in class 1 integron- carrying strains including *arr-2*, *ereC*, *aadA1*, *cmlA5* and *arr2*, *cmlA5*. The integron gene cassette array *arr2*, *cmlA5* was the most prevalent type and identified in 42 (79.2%) of *intI1*-positive *A. baumannii* strains. MBL genes including *bla*_VIM_ and *bla*_IMP_ were detected in 65/68 (95.6%) and 58/68 (85.3%) of *A. baumannii* isolates respectively, out of which 26/28 (92.8%) and 27/28(96.4%) % of the isolates from ICUs and 39/40 (97.5%) and 31/40 (77.5%) of the isolates from the burn wards contained *bla*_VIM_ and *bla*_IMP_ genes respectively (Fig. 3). None of the *A. baumannii* strains studied contained the *bla*_NDM-1_ gene. The co-carriage of three genes *intI1*, *bla*_VIM_ and *bla*_IMP_ was observed in 23/28 (82.1%) and 29/40 (72.5%) of isolates from the ICUs and the burn wards respectively (Table 3).

**Table 3:** Distribution of MBLs and class 1 integrons genes in *A. baumannii* isolates from environmental samples in burn wards and ICUs^†^

Isolates N (%)	Source	*bla*_VIM_	*bla*_IMP_	*bla*_NDM−1_	*intI1*	*intl CS*
23 (82.2)	ICUs (N = 28)	+	+	-	+	+
2 (7.1)		+	+	-	-	-
1 (3.6)		+	-	-	-	-
2 (7.1)		-	+	-	-	-
29 (72.5)	Burn wards (N = 40)	+	+	-	+	+
4 (10.0)		+	+	-	-	-
1 (2.5)		+	-	-	+	+
4 (10.0)		+	-	-	-	-
2 (5.0)		-	-	-	-	-

^†^ICUs: Intensive care units

## Discussion

The persistence of *A. baumannii* in the hospital environment leads to this bacterium facing selective pressure caused by antibiotics [24]. This feature, along with the high potential in acquiring antibiotic resistance genes, leads to the emergence and spread of MDR *A. baumannii* strains in the hospital environment. MDR *A. baumannii* causes life-threatening infections with high mortality, especially in immunocompromised patients admitted to the ICUs [25]. Our study showed that 22.9% of 297 environmental samples collected from the surfaces of studied hospitals were positive for *A. baumannii*, which is somewhat higher compared to other similar studies [10, 26]. The hygiene of the hospital environment is multi-factorial and complex and depends on various variables, including surface type, imprecise disinfectant contact time, excessive dilution of disinfectant solutions, and other factors. Studies indicate that the manual disinfection of surfaces in hospitals is suboptimal, and despite the use of proper disinfection protocols, about 5 to 30% of surfaces remain contaminated [27]. The high-level surface contamination with *A. baumannii* in the present study may be related to the mentioned factors or it may be due to the lack of accurate knowledge of the characteristics of this bacterium during the disinfection of the hospital environment. In a study investigating the persistence characteristics of *A. baumannii*, it was shown that after four rounds of manual disinfection with a bleach solution, 25% of the rooms were still contaminated with *A. baumannii* [28]. The study of the resistance pattern indicated that, all (100%) *A. baumannii* isolates from burn wards and ICUs were MDR and the resistance rate to imipenem in ICUs was also 100%. In a study in Brazil, 80% of *A. baumannii* isolates from ICU environment and 80.4% of isolates from ICU patients at the same hospital were imipenem resistance [24]. Consistent with the present study, in other studies conducted in Iran and Asian countries, the majority of *A. baumannii* isolates from clinical and environmental samples in ICUs were MDR, and resistance to carbapenems has been reported with high frequency [10, 29]. Likewise, all studied strains of *A. baumannii* were sensitive to colistin, and tigecycline. Since the environmental isolates of *A. baumannii* are considered as the source of outbreaks in hospitalized patients, the susceptibility of these strains to last resort antibiotics such as colistin and tigecycline is of great importance. In some studies, conducted in Iran, resistance to the above antibiotics has been reported among clinical resistant *A. baumannii* isolates from ICUs [29]. In addition, the resistance to minocycline and ampicillin-sulbactam in the studied isolates was 7.1%, which is similar to the study conducted by Ying et al. in *A. baumannii* isolates from the environment of the ICUs [10]. Enzymatic inactivation of carbapenems is the most important mechanism of carbapenem resistance in *A. baumannii* and is usually carried out by carbapenemases, which are commonly associated with transmissible genetic elements such as integrons [30]. MBLs and OXA-type carbapenemases are the most prevalent carbapenemases in *A. baumannii* [6]. The results of the analysis of MBL genes indicated that *bla*_VIM_ and *bla*_IMP_ genes were highly prevalent in the studied isolates although *bla*_NDM−1_ was not detected in the mentioned isolates. In agreement with our results, in study conducted by Amin et al. in Ahvaz in the southwest of Iran *bla*_VIM_ has been reported as the most common MBL encoding gene followed by *bla*_IMP_, among clinical isolates of *A. baumannii* [31]. Our previous study in *A. baumannii* isolates from burn wound infection, also indicated *bla*_VIM_ was the most common MBL [15]. Although this finding is contrary to the results obtained in the northwest of Iran in which no clinical *A. baumannii* isolates from the hospital wards and ICUs carried *bla*_VIM_ gene, and the *bla*_NDM−1_ has been reported as the most common MBL gene in the mentioned isolates [32]. The *bla*_NDM−1_ has also been reported as the predominant MBL in *A. baumannii* strains isolated from neonatal sepsis in India [33]. The findings indicate that the pattern of MBL genes in *A. baumannii* strains in various geographical regions and distinct clinical settings is different from each other. Various studies have identified integrons as one of the most important factors in the acquisition of antibiotic resistance genes in *A. baumannii* strains [34]. In particular, the relationship between integrons and MBL genes has been reported in *A. baumannii* strains [15]. In the present study, a high percentage of *A. baumannii* isolates, especially strains isolated from ICUs, carried class 1 integron. Compared to the results obtained in the study conducted in Africa on the *A. baumannii* isolates from the extra-hospital environment which lacked integrons class 1, this finding shows the very high potential of our studied strains to acquire and expand antibiotic resistance genes [35].

## Conclusion

The findings of the present study showed that the isolation rate of MBL producing CRAB was relatively high in the environmental surface of burn wards and ICUs, which can be considered as a potential source of outbreaks in hospitalized patients. Applying standard hospital infection prevention and control measures including hand hygiene, intense monitoring and better environmental disinfection can be the effective activities to prevent the outbreak of MDR *A*. *baumannii* in the hospitals.

### Limitations

The most important limitation that can be expressed in this study is the lack of investigation of *A. baumannii* strains isolated from clinical samples and comparison with environmental isolates in the studied hospitals.

## Data Availability

The datasets analyzed during the current study are available from the corresponding author on reasonable request.

## Abbreviations

BHI: Brain Heart Infusion broth
CLSI: Clinical and Laboratory Standard Institute
CRAB: Carbapenem-resistant *A. baumannii*
EUCAST: European Committee on Antimicrobial Susceptibility Testing
ICUs: Intensive care units
MBL: Metallo-β-lactamase
MDR: Mmultidrug-resistant
MICs: Minimum inhibitory concentrations
NDM: New Delhi β-lactamase
SPSS: Statistical Package for Social Sciences software
